# Factors Related to COVID-19-Preventive Behaviors among Flight Attendants

**DOI:** 10.3390/ijerph191610201

**Published:** 2022-08-17

**Authors:** Chia-Yi Fang, Chia-Jung Hu, Yih-Jin Hu

**Affiliations:** 1 Department of Health Promotion and Health Education, National Taiwan Normal University, Taipei City 106, Taiwan; 2 Department of Nursing, Da Yeh University, Dacun, Changhua 515, Taiwan

**Keywords:** COVID-19, health belief, preventive behavior, flight attendant, occupational health

## Abstract

The health and safety of airline employees have been important issues during the 2019 coronavirus disease (COVID-19) pandemic. The exposure of flight attendants to passengers with severe acute respiratory syndrome coronavirus 2 infection without protective equipment is known to cause in-flight transmission and the worldwide spread of the virus. However, very few studies have focused on flight attendants’ COVID-19-preventive behaviors and related factors. This cross-sectional study was performed to investigate relationships between COVID-19-preventive behaviors and relevant factors in a convenience sample of flight attendants. In total, 261 flight attendants working for two international airlines were recruited. A self-developed questionnaire was used to assess participants’ COVID-19 information-seeking behavior, perceived susceptibility, severity, self-efficacy, and preventive behaviors. Analysis of variance and Pearson’s correlation analysis were performed to analyze COVID-19 preventive behaviors according to socio-demographic and COVID-19-related factors. Multiple regression analysis was used to predict the flight attendants’ COVID-19-preventive behaviors. The factors that primarily influenced participants’ COVID-19-preventive behaviors were gender (women; β = 0.17, *p* < 0.001), information-seeking behavior (β = 0.39, *p* < 0.000), perceived severity (β = 0.130, *p* < 0.05), and self-efficacy (β = 0.17, *p* < 0.05). These factors explained 30.6% of the variance in COVID-19-preventive behaviors among flight attendants. Identification of the preventive behaviors performed by this population is important, as it aids the development of strategies to enhance such behaviors, thereby reducing the in-flight spread of COVID-19.

## 1. Introduction

The 2019 coronavirus disease (COVID-19), first reported in China in December 2019, is caused by severe acute respiratory syndrome coronavirus 2 (SARS-CoV-2). COVID-19 is transmitted rapidly from human to human by droplets, droplet nuclei, and aerosol particles [1]. People with the disease are most contagious for the first 10 days, even when they have no symptoms [2]. Thus, infected asymptomatic air travelers spread SARS-CoV-2 from country to country. The World Health Organization announced the COVID-19 pandemic on 11 March 2020 [3]. As of 13 July 2022, the pandemic had caused more than 554 million cases and 6.35 million confirmed deaths, making it one of the deadliest in history [4].

Cough droplets released by COVID-19-positive passengers can travel four to five seats forward or backward, and about 5–10 people could be infected onboard in the densely packed environment of a flight cabin [5]. Thus, cabin crews and passengers can be infected under pandemic conditions. However, studies show that the best ways to reduce the risk of SARS-CoV-2 infection are vaccination and preventive behaviors, i.e., consistent mask wearing, hand washing, and physical distancing [6,7,8]. Thus, the promotion of these preventive measures among airline crews and passengers is critical.

The engagement in protective behaviors depends not only on the threat of disease, but also on one’s personal ability to do so [9]. Current studies show that individuals who perceive the risk of SARS-CoV-2 infection are more likely to change their health behaviors to avoid the COVID-19 threat [10,11]. In addition, Shahnazi et al. [12] reported that people with high self-efficacy were more likely to adopt COVID-19-preventive behaviors. Self-efficacy is a person’s belief in their ability to cope with life’s difficulties and challenges, and their ability to do so in appropriate ways [13]. Kim and Kim [14] reported other factors that affect preventive behavior, including gender, age, knowledge, and social support.

The media and official public health messages play important roles in the social perception of risk and sharing of information [15]. Social media has rapidly become a crucial communication tool for the generation, dissemination, and consumption of information, including that about the COVID-19 pandemic [16]. Media coverage in Canada was shown to be an important indicator of public health emergency preparedness [17]. Relationships have been identified among social media usage, threat perception, and preventive behavior during the pandemic, similar to the concept reflected by the extended parallel process model (EPPM) developed by communications scholar K. Witte [18]. This model illustrates how individuals react to fear-inducing messages [18]. It has been used to explain how individuals behave to prevent COVID-19, and the results revealed the influence of social media on health-related behaviors [19].

In the wake of the COVID-19 pandemic, airports have become more crowded as passengers return to traveling. On 12 June 2022, the Centers for Disease Control and Prevention lifted the requirement that air passengers traveling to the United States from foreign countries show a negative COVID-19 viral test or documentation of recovery from COVID-19 before they board their flights [20]. However, the risk of being infected and contagious does not change over time. The most important preventive behaviors are physical distancing, face masking, eye protection, hand hygiene, and the following of basic infection control guidelines [6]. Based on the EPPM and previous studies [18,19], we surveyed the status of and correlations between personal information-seeking behavior, perceptions of COVID-19 infection, self-efficacy, and COVID-19-preventive behaviors among flight attendants.

## 2. Materials and Methods

### 2.1. Study Design and Setting

**Figure 1:**
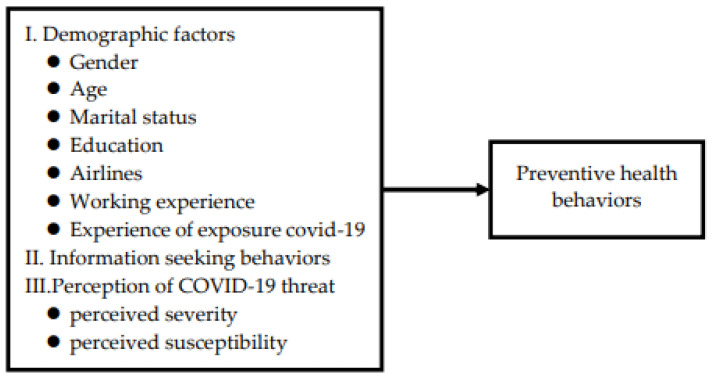
Research framework showing factors potentially associated with preventive health behavior related to COVID-19.

In this study, a cross-sectional approach with structured questionnaire administration was used to assess factors affecting the COVID-19-preventive health behaviors of flight attendants working for two commercial Taiwanese airlines: a Taiwan-based low-cost regional Asian airline and an international airline (see Figure 1). Eligible flight attendants had worked for the airlines for >3 months and understood Chinese. In total, 261 valid questionnaires were returned online. This study was conducted between April and June 2022.

### 2.2. Ethical Considerations

This study was reviewed and approved by the Ethics Committee of En Chu Kong Hospital, Taiwan (ECKIRB1110302). The questionnaire was prepared using Google Forms. We recruited potential participants from the two airlines and distributed the questionnaire via email and social media communication networks. After the participants had provided informed consent to study participation, they completed the anonymous online questionnaire.

### 2.3. Measurements

The questionnaire solicited information about participants’ sociodemographic characteristics, COVID-19 information-seeking behavior, perceived threats (severity and susceptibility), self-efficacy, and COVID-19-preventive behaviors. The scales were self-developed and based on studies related to the EPPM model and other relevant topics [12,18]. The content validity index of the questionnaire was good (>0.9).

#### 2.3.1. Sociodemographic Characteristics

The participants were asked to indicate their gender, age (years), marital status (single, married/cohabitating, divorced/separated), education (bachelors/masters), airline (regional Asian/international), work experience (years), have had COVID-19 on duty (yes/no), have had close contact with COVID-19 cases on duty (yes/no).

#### 2.3.2. Information-Seeking Behavior

Participants’ COVID-19 information-seeking behavior was assessed with two items scored using a 5-point Likert scale (1, strongly disagree; 2, disagree; 3, neutral; 4, agree; 5, strongly agree). Total scores ranged from 2 to 10 points, with higher scores indicating engagement in more COVID-19 information-seeking behavior.

#### 2.3.3. Perception of a COVID-19 Threat

Participants’ perception of (thoughts and feelings about) a COVID-19 threat was measured using severity and susceptibility subscales. Each subscale comprised three items scored using a 5-point Likert scale (range, 1 (strongly disagree) to 5 (strongly agree)), with total scores ranging from 3 to 15 points and higher scores indicating greater perceived severity of or susceptibility to COVID-19 (both Cronbach’s alpha = 0.85).

#### 2.3.4. Self-Efficacy

Self-efficacy was measured as participants’ confidence in their ability to perform COVID-19-protective behaviors using four items scored on a 5-point Likert scale (range, 1 (strongly disagree) to 5 (strongly agree)). Total scores ranged from 4 to 20 points, with higher scores indicating greater self-efficacy for COVID-19–preventive behavior (Cronbach’s alpha = 0.87).

#### 2.3.5. COVID-19-Preventive Behavior

Participants’ COVID-19-preventive behavior (preventive actions undertaken during daily routines, i.e., hand washing, wearing of personal protective equipment, and social distancing) was measured using four items scored on a 5-point Likert scale (range, 1 (strongly disagree) to 5 (strongly agree)). Total scores ranged from 4 to 20 points and reflect hand hygiene, the avoidance of face touching, covering of the mouth and nose, the maintenance of physical distance from others, mask-wearing, and the following of a healthy lifestyle. Higher scores indicate engagement in more COVID-19-preventive behaviors (Cronbach’s alpha = 0.72).

### 2.4. Statistical Analysis

The SPSS statistical software package (ver. 22.0; IBM Corporation, Armonk, NY, USA) was used for data analysis and to generate descriptive statistics. We used Pearson’s correlation analysis to explore the relationships between COVID-19-preventive behaviors and potentially related variables. Single-factor analysis of variance was performed to investigate COVID-19-preventive behavior scores according to sociodemographic variables. Multiple regression analysis was conducted to identify factors predicting COVID-19-preventive behavior. The *p* values < 0.05 were considered to be significant.

## 3. Results

In total, 261 flight attendants working for the two airlines were recruited. Their sociodemographic characteristics are shown in Table 1. Most (*n* = 220; 84.3%) of the flight attendants were women; 52 (19.9%) were aged <40 years, 61 (23.4%) were aged 41–50 years, and 148 (56.7%) were aged 51–65 years. Seventy (26.8%) participants were single, 159 (60.9%) were married or cohabitating, and 32 (12.3%) were divorced or separated; 234 (89.7%) had bachelor’s degrees and 27 (10.3%) had master’s degrees. Twenty-four (9.2%) flight attendants worked for the regional Asian airline and 237 (90.8%) worked for the international airline; 83 (31.8%) participants had <10 years of work experience and more than half (*n* = 178; 68.2%) had >10 years work experience. Ninety (7.3%) participants had had COVID-19, and 197 (41%) had had close contact with COVID-19 cases during duty (Table 1).

Mean preventive behavior scores according to sociodemographic characteristics ranged from 16.26 ± 2.46 to 17.38 ± 2.24. These scores were higher among women with >10 years of work experience (17.28 ± 2.09 vs. 17.38 ± 2.24; Table 1).

Table 2 shows the information-seeking behavior, perceived severity, perceived susceptibility, self-efficacy, and preventive behavior scores. Mean scores ranged from 3.16 ± 1.08 to 4.48 ± 0.59 (neutral to strongly agree). The highest score was for preventive behavior. Two of the highest scores were for behavior scale items 1 (“I would wear a face mask if I had a fever or respiratory symptoms”; 4.33 ± 0.80) and 4 (“I would not go to the hospital during the COVID-19 pandemic if it is unnecessary”; 4.48 ± 0.59). The mean perceived severity and susceptibility scores were 3.16 ± 1.08 and 3.86 ± 0.89 (neutral to agree), respectively. The lowest scores were for susceptibility items 1 (“I am more easily infected with coronavirus than others due to my job.”; 3.16 ± 1.08) and 3 (“If my co-workers became infected with coronavirus, I think I would become infected too”; 3.24 ± 0.87).

In addition to gender, preventive behavior scores correlated significantly with work experience and information-seeking behavior, perceived severity, and self-efficacy scores (Table 3). The regression model result was significant (F = 23.92, *p* < 0.001). These factors predicted 30.6% of the total variance in preventive behavior. Preventive behaviors correlated positively with gender (β = 0.17, 95% CI = 0.41–1.63), information-seeking behavior (β = 0.09, 95% CI = −0.02–0.94), perceived severity (β = 0.13, 95% CI = 0.56–0.99), and self-efficacy (β = 0.17, 95% CI = 0.06–0.26), but not with work experience (β = 0.09, 95% CI = −0.02–0.94) or perceived susceptibility (β = 0.17, 95% CI = 0.06–0.26). Among the predictor factors, the information-seeking behaviors play an important role in COVID-19 preventive behaviors in the regression model (β = 0.39, *p* = 0.000) (Table 4).

## 4. Discussion

Most participants in this study had strong health beliefs and behaviors related to COVID-19, such as the good seeking of information from the government and media, self-efficacy, and COVID-19-preventive behavior. The factors predicting preventive behaviors were gender (women), work experience, information-seeking behavior, perceived severity, and self-efficacy.

The flight attendants’ good seeking of information from the government and media, self-efficacy, and COVID-19 prevention may be attributable to international aviation norms. Airlines focus more on safety and health than general industry, and they hold regular annual in-flight medical training courses. Most of the flight attendants who participated in this study had university degrees; people with higher education levels are more likely to cooperate with the government and undertake more preventive behaviors [21].

Women had higher preventive behavior scores than men in this study, as reported previously [22]. Women who are older, poorer, or in worse health conditions have been shown to perceive a greater contagion risk, be more concerned about COVID-19, perceive the pandemic as a very serious health problem, and agree with restraining measures [23,24]. In this study, participants with more (>10 years) work experience had higher preventive behavior scores. Work experience is proportional to age; senior employees and their family members are older. Due to the high morbidity and mortality of COVID-19 for older subjects, the orders reflect a greater personal health threat from COVID-19 for these subjects, who have been found to be self-disciplined in adhering to COVID-19 prevention rules and procedures [25,26,27]; our study yielded similar findings.

Participants with higher information-seeking behavior, perceived severity, and self-efficacy scores exhibited more preventive behaviors. These results reflect the influence of media exposure, which increases moderate levels of fear and self-efficacy, influencing people to adopt preventive behaviors according to COVID-19 guidelines [28,29]. However, some participants in this study were not highly threatened by COVID-19, and perceived susceptibility was not correlated with preventive behaviors. These findings may reflect participants’ perception that COVID-19 is like influenza and their fatigue by the threat. That also reflects the fact that when the participants had a close contact rate with COVID-19 cases on the duty, only a few people were infected with COVID-19. However, the perceived severity of COVID-19 strongly influenced the flight attendants’ preventive behaviors.

One study showed that the lack of barriers to COVID-19 preventive measure implementation would result in more behaviors in response to such measures [11]. Thus, the provision of masks and disinfectants and the overcoming of environmental barriers effectively improve compliance and engagement in preventive behaviors [12]. As flight attendants frequently fly from country to country and work in limited aircraft spaces, they are regarded as comprising a high-risk group susceptible to viral infection and transmission [30]. Airlines should continue to provide safety and health protection plans and training and adequate protective equipment for flight attendants, strengthen publicity and health education, and prevent the occurrence of infectious diseases to improve occupational health and safety.

A limitation of this study was its narrow focus. It was a pilot study conducted with a convenience sample of flight attendants working for two international airlines in Taiwan. Thus, the results cannot be generalized to flight attendants worldwide. Most participants were women aged >40 years who worked for an international airline, which may have introduced bias. We recommend the performance of additional research with larger and more diverse samples (i.e., the inclusion of flight attendants working for more international and regional companies and examination of additional related factors such as personal health traits, environmental factors, and personality traits). However, the results of this study provide some important messages that aid airlines’ understanding of flight attendants’ health behavior status. Airlines should strengthen awareness and use these messages to enhance COVID-19-preventive behaviors.

## 5. Conclusions

In this study, we characterized flight attendants’ COVID-19-preventive behaviors and identified some related factors. The flight attendants had very good COVID-19-preventive and information-seeking behaviors. Some passengers may be infected by COVID-19 before boarding and be highly contagious, particularly during long-haul flights. Therefore, the preventive behaviors of flight attendants are critical and necessary. We recommend that governments and airlines issue guidance and provide health-protective education as the COVID-19 pandemic progresses. The provision of accurate health information through media, company and government announcements will aid the improvement of risk perception and the undertaking of preventive behaviors. This approach will help reduce the international spread of COVID-19 and ensure the safety and health of airline staff and passengers.

## Figures and Tables

**Table 1:** Participants’ sociodemographic characteristics (*n* = 261). ** *p* < 0.01.

Variable	Category	N	(%)	Preventive Behavior			Post Hoc Comparison
				Mean	SD	*p*	
Gender	Men	41	15.7	16.26	2.49	0.006 **	b > a
	b. Women	220	84.3	17.28	2.09		
Age (y)	≤30 years	9	3.4	16.00	1.93	0.444	
	b. 31–40 years	43	16.5	17.27	1.79		
	c. 41–50 years	61	23.4	17.21	2.28		
	d. 51–65 years	148	56.7	17.10	2.26		
Marital status	Single	70	26.8	17.01	2.22	0.659	
	b. Married or cohabitation	159	60.9	17.10	2.16		
	c. Divorced or separation	32	12.3	17.43	2.28		
Education	Bachelor’s	234	89.7	17.09	2.22	0.536	
	b. Master’s	27	10.3	17.37	1.86		
Airlines	Asian regional	24	9.2	16.79	1.99	0.438	
	b. International	237	90.8	17.15	2.21		
Working experience	≤10 years	83	31.8	16.56	1.96	0.005 **	b > a
	b. >10 years	178	68.2	17.38	2.24		
Have had COVID-19	yes	19	7.3	16.26	2.30		
	b. no	242	92.7	17.19	2.17		
Have had close cotact with COVID-19 cases	yes	107	41	17.04	2.02		
	b. no	154	59	17.17	2.30		

**Table 2:** Descriptive statistics for the information-seeking behavior, perceived severity, perceived susceptibility, self-efficacy, and COVID-19-preventive behavior scales (*n* = 261).

Variable		Range	Mean	SD
***Information-seeking behaviors***				
Information-1	I automatically seek COVID-19 preventive knowledge from the social media.	2–5	4.08	0.62
Information-2	I continually notice the new COVID-19 policy information from the government.	2–5	4.20	0.65
Information-3	Perceiving a COVID-19 threat.			
***Perception of COVID-19 threat***				
***Perceived severity***				
Severity-1	The impact of coronavirus on my financial security is very serious to me.	1–5	3.46	1.00
Severity-2	The impact of coronavirus on my family/friends is very serious to me.	1–5	3.86	0.89
Severity-3	The impact of being infected with coronavirus is very serious to me.	1–5	3.67	0.87
***Perceived susceptibility***				
Susceptibility-1	I am more easily infected with coronavirus than others due to my job.	1–5	3.16	1.08
Susceptibility-2	I think I will be infected with coronavirus during my work time.	1–5	3.34	0.95
Susceptibility-3	If my co-workers become infected with coronavirus, I think I will become infected with coronavirus too.	1–5	3.24	0.87
***Self-efficacy***				
Efficacy-1	I am confident that I would wear an N95 or medical mask correctly.	1–5	4.20	0.65
Efficacy-2	I am confident that I would wear and take off isolation gowns correctly.	1–5	4.11	0.66
Efficacy-3	I am confident that I would wear and take off gloves correctly.	1–5	4.12	0.65
Efficacy-4	I am confident that I would wash my hands before and after working and contact with passengers.	1–5	3.95	0.76
***Preventive behaviors***				
Behavior-1	I would wear a face mask if I had a fever or respiratory symptoms.	1–5	4.33	0.80
Behavior-2	I always wash my hands and do not touch my eyes, nose, or mouth with unwashed hands.	3–5	4.26	0.65
Behavior-3	I do not go to crowded places during the COVID-19 pandemic.	1–5	4.06	0.88
Behavior-4	I would not go to the hospital during the COVID-19 pandemic if it is unnecessary.	1–5	4.48	0.59

**Table 3:** Correlation analysis variables.

Variable	Range	Mean	SD	1	2	3	4	5
Information-seeking behavior	9–15	12.47	1.37	1				
2. Perceived severity	3–15	10.99	2.44	0.10	1			
3. Perceived susceptibility	3–15	9.73	2.56	0.07	0.27 **	1		
4. Self-efficacy	4–20	16.38	2.33	0.31 **	0.12 *	0.01	1	
5. Preventive behaviors	10–20	17.12	2.19	0.48 **	0.20 **	0.00	0.31 **	1

* *p* < 0.05, ** *p* < 0.01.

**Table 4:** Results of regression analysis of variables predicting flight attendants’ COVID-19-preventive behaviors.

Variable	Coefficients		Multiple Models					Adjusted R^2^	F
	B	SE(B)	Beta	95% CI		t	*p*		
Gender (women)	1.02	0.31	0.17	0.41	1.63	3.28	0.001 **	0.306	23.92 ***
2. Working experience (>10 years)	0.45	0.24	0.09	−0.02	0.94	1.85	0.064		
3. Information-seeking behaviors	0.78	0.11	0.39	0.56	0.99	7.13	0.000 ***		
4. Perceived severity	0.12	0.04	0.13	0.02	0.21	2.58	0.010 *		
5. Self-efficacy	0.16	0.05	0.17	0.06	0.26	3.18	0.021 *		

* *p* < 0.05, ** *p* < 0.01, *** *p* < 0.001.

## Data Availability

The raw data supporting the conclusions of this article will be made available by the correspondence author.
